# Marine algae as emerging therapeutic alternatives for depression: A review

**DOI:** 10.22038/ijbms.2021.54800.12291

**Published:** 2021-08

**Authors:** Kogilavani Subermaniam, Seong Lin Teoh, Yoon-Yen Yow, Yin Quan Tang, Lee Wei Lim, Kah Hui Wong

**Affiliations:** 1 Department of Anatomy, Faculty of Medicine, Universiti Malaya, 50603 Kuala Lumpur, Malaysia; 2 Training Management Division, Ministry of Health Malaysia, 62675 Putrajaya, Malaysia; 3 Department of Anatomy, Faculty of Medicine, Universiti Kebangsaan Malaysia Medical Center, Jalan Yaacob Latif, Bandar Tun Razak, 56000 Kuala Lumpur, Malaysia; 4 Department of Biological Sciences, School of Medical and Life Sciences, Sunway University, 47500 Bandar Sunway, Selangor Darul Ehsan, Malaysia.; 5 School of Biosciences, Faculty of Health & Medical Sciences, Taylor’s University Lakeside Campus, 47500 Subang Jaya, Selangor Darul Ehsan, Malaysia; 6 Neuromodulation Laboratory, School of Biomedical Sciences, Li Ka Shing Faculty of Medicine, The University of Hong Kong, 21 Sassoon Road, Pokfulam, Hong Kong Special Administrative Region, China

**Keywords:** Antidepressants, Complementary medicine, Depression, Microalgae, Neuroinflammation, Neuronal plasticity, Seaweed

## Abstract

Depression is a complex heterogeneous brain disorder characterized by a range of symptoms, resulting in psychomotor and cognitive disabilities and suicidal thoughts. Its prevalence has reached an alarming level affecting millions of people globally. Despite advances in current pharmacological treatments, the heterogenicity of clinical response and incidences of adverse effects have shifted research focus to identification of new natural substances with minimal or no adverse effects as therapeutic alternatives. Marine algae-derived extracts and their constituents are considered potential sources of secondary metabolites with diverse beneficial effects. Marine algae with enormous health benefits are emerging as a natural source for discovering new alternative antidepressants. Its medicinal properties exhibited shielding efficacy against neuroinflammation, oxidative stress, and mitochondrial dysfunction, which are indicated to underlie the pathogenesis of many neurological disorders. Marine algae have been found to ameliorate depressive-like symptoms and behaviors in preclinical and clinical studies by restoring monoaminergic neurotransmission, hypothalamic-pituitary-adrenal axis function, neuroplasticity, and continuous neurogenesis in the dentate gyrus of the hippocampus via modulating brain-derived neurotrophic factors and antineuroinflammatory activity. Although antidepressant effects of marine algae have not been validated in comparison with currently available synthetic antidepressants, they have been reported to have effects on the pathophysiology of depression, thus suggesting their potential as novel antidepressants. In this review, we analyzed the currently available research on the potential benefits of marine algae on depression, including their effects on the pathophysiology of depression, potential clinical relevance of their antidepressant effects in preclinical and clinical studies, and the underlying mechanisms of these effects.

## Introduction

Depression is a common mental disorder and a substantial mental health problem worldwide. The core symptoms of depression include lowering of mood, reduced energy, and decreased activity (1). Besides insomnia or hypersomnia, other manifestations of the disease can include altered appetite or weight, poor concentration, feelings of worthlessness or guilt, and recurrent suicidal thoughts (2). Depression has emerged to be one of the three leading causes of non-fatal health loss in the past three or so decades (3). The prevalence of depression has reached an alarming level, affecting an estimated 350 million people worldwide (4). The lifetime prevalence of depression was 10.8% between 1994 and 2014, with the prevalence particularly high among females (5, 6). Among depressed individuals, 40% had their first episode before the age of 20 years, 50% were aged between 20 and 50, and 10% were over 50. Depression can influence the risk of mortality, as severe depression is strongly associated with suicide. It is estimated 800,000 people die of suicide every year, and approximately 85% of these cases are related to major depression (5, 6).

Various pharmacotherapy and psychotherapy treatments are currently available for the management and treatment of depression. Second-generation antidepressant drugs (e.g., selective serotonin reuptake inhibitors, serotonin, and noradrenaline reuptake inhibitors) and other newer agents (e.g., serotonergic antidepressants, noradrenaline, and dopamine reuptake inhibitors) are considered as the first-line treatments due to their safety and tolerability compared with first-generation antidepressant drugs (e.g., tricyclic antidepressants and monoamine oxidase inhibitors) (7). However, the use of second-generation antidepressants is associated with numerous adverse effects, including dry mouth, gastrointestinal symptoms (nausea, diarrhea, gastric bleeding, and dyspepsia), cardiovascular disturbances (heart rate, QT interval prolongation, hypertension, and orthostatic hypotension), genitourinary symptoms (urinary retention and incontinence), hepatotoxicity, seizures, and weight gain (8). Therefore, it is crucial to identify new efficacious antidepressant treatments with little or no adverse effects. 

Although synthetic compounds have been developed as antidepressant drugs, the use of naturally derived compounds has gained much attention owing to their medicinal properties, general safety, and tolerance (9-12). An increasing number of clinical cases have shown that there is growing interest in phytomedicines among healthcare practitioners and patients. The development of antidepressant drugs from plant origins typically requires a multidisciplinary approach, including but not limited to ethnopharmacological survey, phytochemical and pharmacological studies. Marine algae have been shown to contain polyphenols that have a wide array of therapeutic benefits, including anti-oxidant activity (2, 12, 13-16). The discovery of antidepressant effects of marine algae provides the opportunity for the development of promising treatments for depression (12, 17-21). Given the critical role of oxidative stress and neuroinflammation in the onset and development of depression, marine algae could be a valuable source for the discovery of novel therapeutics for depression. This review summarizes the research on neurobiology of depression, and preclinical and clinical studies of antidepressant effects of marine algae. 

**Understanding the pathophysiology of depression**

*Modulation of monoaminergic neurotransmitters*

Functional insufficiency of monoaminergic neurotransmitters in the brain including noradrenaline (NE), serotonin (5-HT), and dopamine (DA) has been identified as the main underlying cause of depression (22). The discovery of antidepressant drugs in the 1950s resulted in the emergence of the first biochemical hypothesis of depression, which proposed that an alteration in central monoaminergic function was the primary underlying lesion of the disorder (23). This hypothesis was further supported by clinical evidence and animal experiments, in which administration of reserpine, an antihypertensive drug, resulted in the depletion of presynaptic stores of NE, 5-HT, and DA, and also led to a syndrome resembling depression in some patients (24). Conversely, iproniazid, a synthesized compound for treating tuberculosis, was found to attenuate the activity of metabolic enzyme monoamine oxidase (MAO) and elevate extracellular levels of NE and 5-HT in the brain, leading to euphoria and hyperactive behavior in some patients (25).

**Figure 1:**
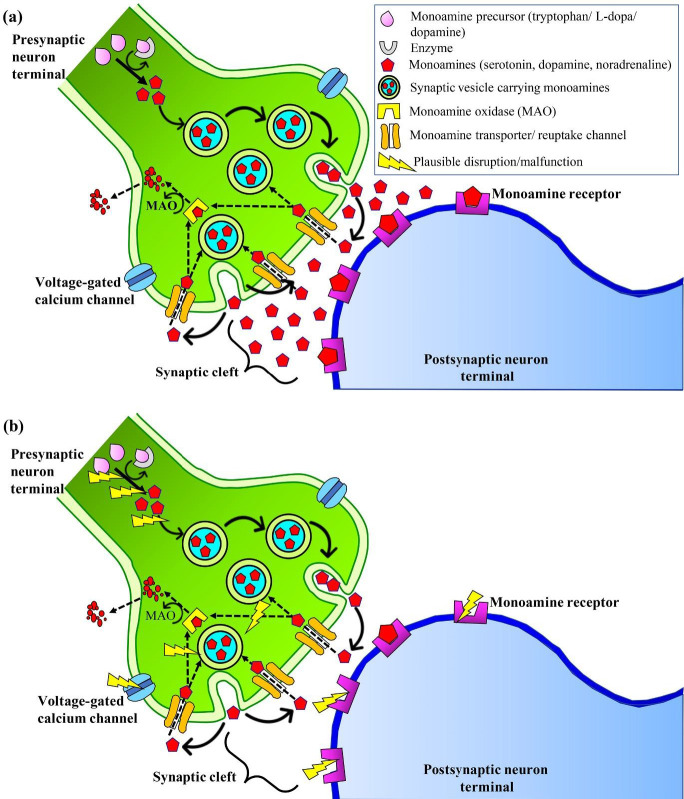
The hypothesis of modulation of monoaminergic transmitters in the pathophysiology of depression. (a) Normal neurotransmission in the monoamine system and (b) Potential disruptions of precursors, enzymes, storage and release of monoamines, monoamine receptors, and exocytosis, leading to a deficiency in neurotransmission. Solid arrows show the course of monoamine synthesis, transportation of monoamine neurotransmitters in the synaptic vesicles, and the release of neurotransmitters into the synaptic cleft. Dashed arrows show the reuptake of monoamine neurotransmitters from the synaptic cleft into the presynaptic neuron for recycling or to be broken down by monoamine oxidase

As noradrenergic, serotonergic, and dopaminergic neurons are brain-derived and project into various regions of the brain including the hippocampus, frontal cortex, and amygdala, it was proposed that the monoaminergic system was responsible for the behavioral symptoms and psychomotor agitation. Furthermore, atypical functions and behavioral changes that contribute to the development of depression may be a result of alterations in the synthesis, storage, or release of neurotransmitters; malfunction of monoamine receptors, enzymes, precursors; or reduced exocytosis (26) (Figure 1). Subsequently, monoamine oxidase inhibitors such as tranylcypromine, phenelzine, isocarboxazid, and others were developed to improve the symptoms of depression (27). Notably, the major role of the monoaminergic system in the etiology and pathophysiology of depression is further manifested by the act of classical antidepressants in restoring the monoamines to normal levels (28).

*Dysregulation of the hypothalamic-pituitary-adrenal (HPA) axis*

**Figure 2:**
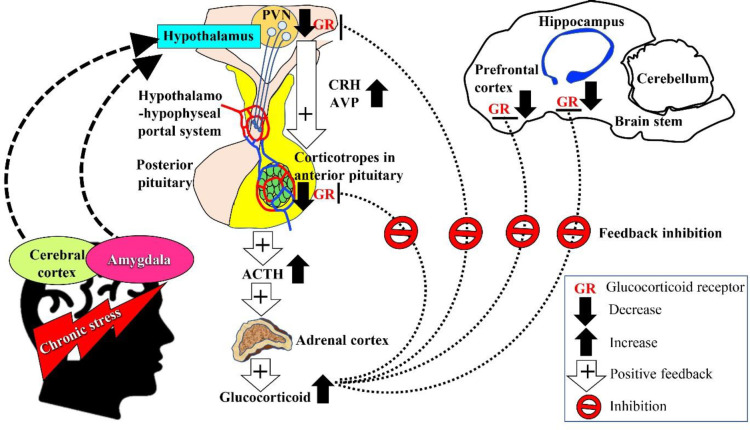
The hypothesis of dysregulation of HPA axis in the pathogenesis of depression. Chronic stress is perceived by the cerebral cortex and amygdala, and transmitted to the hypothalamus (indicated by dashed arrows). Activated PVN releases CRH and AVP into the hypothalamo-hypophyseal portal system. Corticotrophin-releasing hormone and AVP stimulate the corticotropes of the anterior pituitary gland to secrete ACTH into the bloodstream, acting on the zona fasciculata of the adrenal cortex to secrete glucocorticoids. Arrows with plus sign indicate a positive feedback mechanism towards the synthesis and secretion of glucocorticoids into the bloodstream in response to chronic stress. Dotted lines indicate the reduced expression of GR in the prefrontal cortex, hippocampus, PVN, and anterior pituitary gland due to GR-mediated negative feedback loss. The schematic diagram was adapted and modified from Lew *et al*. (11)

The hypothalamic-pituitary-adrenal (HPA) axis comprises a complex set of direct effects and feedback interactions between the hypothalamus, pituitary and adrenal glands to modulate the response to physical, physiological, or psychological stressors (11). The anatomical networks between the amygdala, hippocampus, prefrontal cortex, and hypothalamus facilitate the integration of information related to emotion and cognition in response to stress, which leads to activation of the HPA axis (Figure 2). Upon exposure to stress, neurons in the paraventricular nucleus (PVN) in the hypothalamus increase the synthesis of corticotropin-releasing hormone (CRH), which is transported to the anterior pituitary gland via the hypothalamo-hypophyseal portal system. The CRH then stimulates corticotropes to synthesize proopiomelanocortin, a precursor of adrenocorticotropin hormone (ACTH). Subsequently, arginine vasopressin (AVP) acts synergistically with CRH to stimulate ACTH release into the systemic circulation, causing the release of glucocorticoids from the zona fasciculata of the adrenal cortex. Glucocorticoid cortisol (in humans) and corticosterone (in mice) act on various organ systems redirecting resources to ensure sufficient supply of energy to regulate physiological and behavioral changes. The regulation of glucocorticoid release is mediated via a feedback mechanism involving the less sensitive glucocorticoid receptors in the hippocampus, hypothalamus, and pituitary gland that assist in limiting persistent exposure to catabolic glucocorticoids (11, 29).

The activation of HPA axis in response to acute stress results in time-based beneficial effects, whereas activation in response to chronic stress leads to an imbalance of HPA axis. Hyperfunction of the HPA axis has been shown to be associated with CRH hyperactivity, reduced negative feedback ability, and hypersecretion of glucocorticoids in patients with depression (29). The elevated level of CRH messenger ribonucleic acid (mRNA) was also associated with increased numbers of PVN neurons expressing CRH and downregulation of pituitary CRH receptors in depressed patients. In addition, increased levels of plasma and cerebrospinal CRH, plasma cortisol, and episodes of ACTH secretion were also reported in patients with major depression. Imaging studies revealed reduced hippocampal volume and enlarged pituitary and adrenal glands in patients with major depression. Similarly, preclinical evidence has shown depressive-like behaviors and elevated serum levels of CRH, ACTH, and corticosterone in an animal model of depression induced by unpredictable chronic mild stress (30). Conversely, treatment with antidepressants was found to normalize HPA axis dysregulation-derived changes in depressed patients (31). The mechanisms of HPA axis dysregulation in depression may involve multiple pathways. The glucocorticoid receptor (GR) hypothesis proposes that GR resistance and reduced negative feedback trigger the increase of CRH, ACTH, and glucocorticoids. The hypothesis of CRH activation suggests the involvement of multiple feedback cycles including the downregulation of GR due to excess glucocorticoids in circulation, which leads to the hyperactivity of the HPA axis. Moreover, CRH might be proficient in enhancing its own biosynthesis in PVN. Therefore, prolonged activation of the HPA axis upregulates the amygdaloid CRH system (29).

*Neuroplasticity involving brain-derived neurotrophic factor (BDNF)*

**Figure 3:**
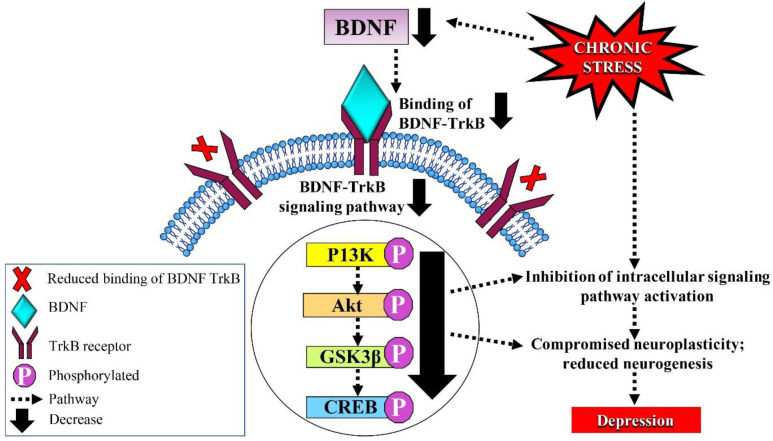
The hypothesis of neuroplasticity involving BDNF in the pathogenesis of depression. Chronic stress leads to a decrease in hippocampal BDNF expression, causing a reduction in the binding of BDNF to TrkB receptors needed for activation of the BDNF-TrkB signaling pathway (indicated by solid arrows). This results in compromised neuroplasticity, and reduced neuronal survival rate, dendritic growth, spine density, synaptogenesis, and maturation of neurons

The neurotrophic hypothesis of depression suggests that neuroplasticity is a central factor in the evolution of depression and the clinical response toward antidepressant drugs. Brain-derived neurotrophic factor, a family of neurotrophic factors, has a central role in the survival and differentiation of different populations of neurons in the developing brain, including maintaining a high level of presynaptic neurotransmitter release, excitatory and inhibitory synaptic communication, and activity-dependent neuroplasticity (32). In addition, BDNF has a key role in neuroplasticity related to learning, memory, and emotion. Brain-derived neurotrophic factor was also found to promote neuronal survival rate, dendritic growth, spine density, synaptogenesis, and maturation of neurons, all of which are crucial for the development of learning and adaptation processes that are found to be decreased in depression (33). Preclinical and clinical studies have revealed that chronic stress leads to hippocampal BDNF depletion and reduced binding of BDNF to tyrosine receptor kinase B (TrkB) (Figure 3). Reduced level of serum BDNF has been shown to be associated with depressive-like symptoms and behaviors in clinical studies (34). Preclinical findings have indicated an association between stress-induced depressive-like behaviors and a reduction in the level of hippocampal BDNF (35).

Chronic administration of antidepressants has been found to elevate the expression of BDNF mRNA in rat hippocampus via 5-hydroxytryptamine receptor 2A (5-HT_2A_) and β-adrenoceptor subtypes (36). Brain-derived neurotrophic factor is released as a mixture of pro and mature BDNF in an activity-dependent manner, in which the former binds with pan neurotrophin receptor (p75 NTR) to induce apoptosis and facilitate long-term depression (37), whereas mature BDNF binds to TrkB to promote cell survival and increase neuronal spine complexity (38). Neurogenesis, a process involving cell division, migration, and differentiation, was found to be enhanced by BDNF in the hippocampus, altering cell survival and proliferation, and contributing to the antidepressive mechanism (37). Neurogenesis has been shown to occur in subventricular and subgranular zones of the dentate gyrus (21). Therefore, enhanced hippocampal neurogenesis via elevated BDNF and BDNF-TrkB signaling-derived cognitive alterations might show great potential as therapeutic targets for the discovery of novel antidepressants (21, 37).

*Neuroinflammation*

Recent preclinical and clinical evidence have repeatedly suggested the involvement of neuroinflammation and cytokines as one of the key factors contributing to the pathogenesis of depression. Studies have shown the interaction of neuroinflammation with three other neurobiological correlates of depression, namely monoamine deficiency, suppression of neurogenesis, and HPA axis dysregulation (39, 40). Furthermore, central and peripheral pro-inflammatory cytokines; tumor necrosis factor-alpha (TNF-α), interleukin-6 (IL-6), and interleukin 1β (IL-1β) were reported to have a strong correlation with depression (40). Exposure to cytokines and lipopolysaccharide (LPS)-induced neuroinflammation resulted in the development of depressive-like symptoms including mood alteration, stress reaction, cognitive impairment, reduced locomotor activity, and sleep disorder (17). Exposure to LPS significantly increased the expression of proinflammatory cytokines IL-1β, IL-6, TNF-α, and inflammatory mediators including inducible- and neuronal nitric oxide synthases (iNOS and nNOS), cyclooxygenase-2 (COX-2), and COX-2 mRNA expression via activation of nuclear factor kappa B (NF-κB) in the hippocampus of an animal model of depression (17). Conversely, antidepressant treatments exhibited antidepressant-like effects and were accompanied by a reduction in brain inflammatory cytokines and mediators (IL-1β, IL-6, TNF-α, iNOS nNOS, and COX-2) and COX-2 mRNA expression as well as reduced phosphorylation of NF-κB p65 (17).

The contribution of neuroinflammation in the pathogenesis of depression was further supported by clinical trials, in which plasma cytokines and acute-phase protein concentrations in the blood were elevated in patients with major and treatment-resistant depression (41). Healthy individuals exposed to stressful life events were shown to have impaired immune function at the cellular level, whereas depressed patients exposed to the events showed increased levels of IL-6 and acute phase C-reactive protein (CRP) (42). An upregulation of interleukin-1 (IL-1), a regulator of serotonin (5-HT) transporter gene, and HPA axis activity contributed to the generation of stress-like effects on immune function and altered monoamine neurotransmitters. In addition, pretreatment with exogenous cytokines such as interleukin-2 (IL-2) and interferon-α (IFN-α) in healthy individuals resulted in the development of depressive-like symptoms (39). Nitric oxide (NO), synthesized from L-arginine via nitric oxide synthase (NOS) during neuroinflammation, plays a major role in the pathogenesis of major depression. Further, there is an elevation of plasma NO metabolites in suicidal and depressed patients (43). Inhibitors of NOS (7-nitroindazole and methylene blue) have been demonstrated to decrease NO production and promote antidepressant effects in a rat model of depression study (44).

**Figure 4:**
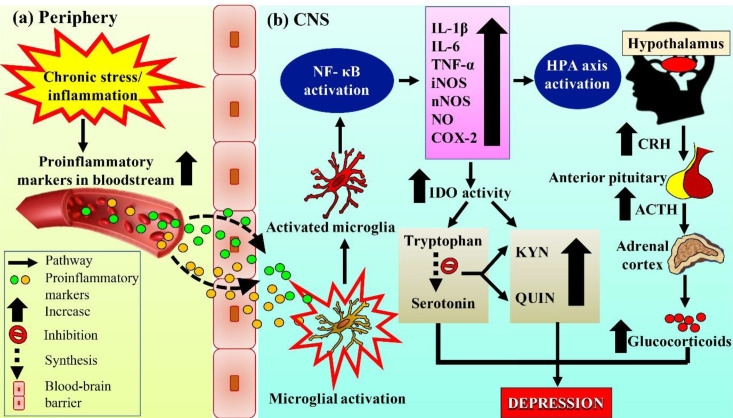
The hypothesis of neuroinflammation in the pathogenesis of depression. (a) Chronic stress or inflammation leads to the expression of proinflammatory markers in the periphery. Dashed arrows indicate transportation of the markers through the BBB into CNS to activate microglia. (b) Activated microglia produce proinflammatory cytokines and mediators via activation of NF-κB. The neuroinflammatory response enhances HPA axis hyperactivity, IDO activity, and release of CRH, ACTH, and eventually glucocorticoids. Simultaneously, up-regulated proinflammatory cytokines and mediators also enhance IDO activity, inhibit the synthesis of serotonin from tryptophan (indicated by a dotted arrow) and increase the production of KYN and QUIN

Although the exact mechanism of the involvement of neuroinflammation in the pathogenesis of depression remains unclear, accumulating evidence suggests the existence of crosstalk between chronic stress-induced intracellular reactive oxygen species (ROS) generation in brain tissues and disruption of synaptic and non-synaptic communication, leading to neuroinflammation and cell death (39, 45) In addition, stress-induced HPA axis dysfunction is thought to be further reinforced by chronic inflammation involving increased peripheral inflammatory markers that are capable of crossing the blood-brain barrier, thus activating microglia in the early manifestation of symptoms of depression (Figure 4). Furthermore, continuous production of proinflammatory cytokines and mediators during neuroinflammation enhances intracellular ROS production as a result of HPA axis hyperactivation, indicating a vicious cycle between the HPA axis and neuroinflammation (39, 40, 45, 46). Upregulation of neuroinflammation is involved in the development of depression by stimulating indoleamine 2,3-dioxygenase (IDO) that leads to increased production of toxic tryptophan catabolites, degradation of serotonin, production of kynurenic acid (KYN) and quinolinic acid (QUIN), and proinflammatory mediators. Quinolinic acid also acts as a neurotoxin by altering the integrity of the blood-brain barrier (BBB) (47). However, even though the inflammatory-mediated changes in the central nervous system are momentary and vanish after removal of the stimulus, these cytokines may play a pivotal role in producing depressive-like symptoms, which could be targeted by anti-inflammatory compounds as a therapy for depression (47). 

**Marine algae in the treatment of depression**

Approximately 50% of depressed patients have been reported to have used some form of complementary and alternative medicine (48). Marine algae have been used as food, fertilizer, and as a source of medicines since ancient times, and more recently, as a raw material in food, cosmetics, and the pharmaceutical industry, and as a medicine (49). Marine algae consist of a diverse group of large macro- and micro-algae. Marine microalgae include diatoms, dinoflagellates, green algae, and blue-green algae (cyanobacteria), while macroalgae commonly referred to as seaweed, are a taxonomically diverse group of plants classified into Rhodophyceae (red), Chlorophyceae (green), and Phaeophyceae (brown). Marine algae can grow rapidly and do not require arable land or freshwater for their cultivation, warranting their broader use in pharmaceutical and nutraceutical industries (50). Moreover, marine algae are rich in secondary metabolites for therapeutic applications, some of which are uncommon in terrestrial plants. The compounds, namely phlorotannins, alginates, fucoidan, sargaquinoic acid, sulfated polysaccharides, and carotenoids possess anticoagulant, antiviral, anti-oxidant, anti-allergic, anticancer, anti-inflammatory, anti-obesity, and neuroprotective activities (51). In this review, we attempt to highlight the antidepressant effects of 14 species of marine algae in preclinical and clinical studies. A summary of the findings is shown in Table 1.

***Preclinical studies***

*Arthrospira platensis Gomont*

*Arthrospira platensis*, previously known as *Spirulina platensis* and commonly referred to as *Spirulina,* is a true puree of a filamentous, spiral-shaped, blue-green freshwater microalga. It is widely cultivated in Korea (34) and has been isolated in the Chenghai lake in China, soda lakes in East Africa, and subtropical alkaline lakes (61). *Spirulina,* the dried biomass of *A. platensis*, has been branded as a superfood enriched with nutrients including proteins, carbohydrates, polyunsaturated fatty acids, sterols, minerals, and vitamins (62). Kim *et al*. reported the antidepressant effect of *Spirulina* in a mouse model of depression induced by forced swim test (FST). It was found that oral administration of hydrolyzed *Spirulina *extract by malted barley at 10 ml/kg body weight/day for 2 weeks significantly reduced depressive-like symptoms (34). The decrease in immobility time in FST was associated with a reduction of blood urea and nitrogen (BUN), and lactate dehydrogenase (LDH) release. However, the hydrolyzed extract had no effect on creatine kinase, glucose, total protein, and albumin levels (34). 

A study reported the antidepressant effects of *S. platensis* in rat and mouse models of depression evaluated by FST, tail suspension test (TST), clonidine-induced aggression behavior, levodopa (L-DOPA)-induced hyperactivity and aggressive behavior, and 5-HT-induced head twitches (52). Daily oral administration of *S. platensis *at 100, 200, or 400 mg/kg body weight/day for 7 days ameliorated depressive-like behaviors with a significant reduction of immobility time in FST and TST. The behavioral tests were conducted 1 hr after oral administration of *S. platensis* or intraperitoneal injection of standard drugs (15 mg/kg imipramine or 2.5 mg/kg lorazepam) on day 7 except for FST on day 8. It was found that *S. platensis *reverted the disrupted neurotransmission in depression (52). There were significant increases in the frequency of 5-HTP-induced head twitches, clonidine-induced aggression, L-DOPA-induced hyperactivity, and aggressive behavior, suggesting that *S. platensis* had a role in enhancing serotonergic, noradrenergic, and dopaminergic pathways in depression (52, 63).

Additionally, pretreatment with *S. platensis* significantly increased the levels of SOD and catalase, and decreased the rate of lipid peroxidation, suggesting its potent anti-oxidant activity (52). Oxidative damage is involved in the pathogenesis of depression characterized by an increase of lipid peroxidation products, deoxyribonucleic acid (DNA) damage, and ROS production, leading to the destruction of phospholipids and neuronal membrane viscosity. The events cause perturbations of serotonergic and catecholaminergic neurotransmission (12, 23). Furthermore, malondialdehyde (MDA) directly inhibits the binding of serotonin to its receptor, altering the intricate balance between serotonin metabolism and oxidative stress. Therefore, the anti-oxidant mechanisms of *S. platensis* might have contributed to its antidepressant effects (52). 

Moradi-Kor *et al.* demonstrated the therapeutic effects of *S. platensis* against adolescent stress in BDNF alterations, and molecular and morphological remodeling in the basolateral amygdala of adult female rats induced by restraint stress. Adolescent stress increased the oxidative stress parameters and decreased the BDNF level, apical dendritic length, and branch points of pyramidal neurons (21). Oral administration of *S. platensis* at 200 mg/kg body weight/day for 15 days alleviated oxidative stress, increased ferric reducing anti-oxidant power (FRAP), and reduced MDA level in the basolateral amygdala (21).

Stressful experience in the pre-pubertal period has been found to alter the response to stressors and increases the vulnerability to stress-related disorders in adulthood (64). Substantial remodeling in the prefrontal cortex, hippocampus, and amygdala due to chronic stress could contribute to mood disorders such as depression and anxiety (64, 65). Interestingly, Moradi-Kor *et al.* also showed that adolescent stress decreased BDNF and TrkB mRNA expressions in the basolateral amygdala, indicating the potential involvement of BDNF and its receptors in stress-induced depressive disorders (21). 

*Botryococcus braunii* Kützing

*Botryococcus braunii *is a pyramid-shaped green colonial microalga that contains triterpenes. It is found in freshwater lakes, brackish lakes, reservoirs, and ponds worldwide (19, 66). Sasaki *et al.* reported the antidepressant effects of *B. braunii* ethanol extract in the FST-induced mouse model of depression. Daily oral administration of *B. braunii* ethanol extract at 100 mg/kg body weight/day for 14 days followed by FST on days 1, 2, 6, 10, and 14 ameliorated depressive-like behaviors with decreased immobility in the FST (19). There was an upregulation of gene expression associated with energy metabolism (polyribonucleotide nucleotidyltransferase 1, Pnpt1), dopamine production (arginine/serine-rich coiled-coil 1, Rsrc1), and neurogenesis (short stature homeobox 2, Shox2; paired-like homeodomain transcription factor 2, Pitx2; teashirt zinc finger family member 1, Tshz1; LIM homeobox protein 9, Lhx9). mRNA expression levels of BDNF, tyrosine 3-monooxygenase (TH), and pyruvate carboxylase (PC) were also upregulated. They postulated that the enhanced energy production may have attributed to the modulation of neurogenesis and enhancement of dopaminergic function, indicating antistress and antidepressant effects (19).

*Chlorella vulgaris* Beijerinck, M.W.

*Chlorella vulgaris *is a freshwater unicellular green microalga that contains high level of chlorophyll (67). It is widely cultivated in Germany, Japan, and Taiwan (68). *C. vulgaris* has been shown to have various pharmacological properties including anti-oxidant, anti-inflammatory, and anti-aging activities (67). Recently, Soetantyo and Sarto have investigated the effects of cultivated and commercially sold *C. vulgaris* in a rat model of depression induced by chronic unpredictable mild stress (CUMS) (53). Daily oral administration of *C. vulgaris* at 360 mg/kg body weight/day for 14 days following 42 days of stress induction ameliorated the depressive-like behaviors with reduced immobility time in FST, increased exploratory behavior in OFT, and restored sucrose preference in SPT. These results suggest its neuroprotective effects against CUMS. However, pretreatment of *C. vulgaris* did not appear to restore the size of hypertrophied adrenal glands (53).

*Haematococcus pluvialis* Flotow

*Haematococcus pluvialis *is a freshwater unicellular green microalga found in temperate regions globally (69). It has commercial potential owing to its ability to accumulate massive amounts of red ketocarotenoid pigment, namely astaxanthin (AST; 3,3′-dihydroxy-β, β-carotene-4,4′-dione) (70). Jiang *et al.* investigated the effects of AST against LPS-induced neuroinflammation and depressive-like behaviors in a mouse model of depression. Daily oral administration of AST at 20, 40, or 80 mg/kg body weight/day for 7 consecutive days followed by intraperitoneal injection of LPS at 0.83 mg/kg body weight on day 7 restored the LPS-induced immobility time in FST and TST, indicating a reduction of depressive-like behaviors (17).

Restoration of neurochemical alterations of proinflammatory cytokines (IL-1β, IL-6, and TNF-α), inflammatory mediators (iNOS, nNOS, NO, and COX-2), COX-2 mRNA expression, and nuclear factor kappa B (NF-κB) in the hippocampus and prefrontal cortex contributed to the antidepressant-like and neuroprotective effects of AST (17). In addition, the ability of AST to locate itself within the phospholipid membrane or its surface, as well as to cross the BBB, may also be responsible for its neuroprotective effects (71-73).

Similarly, Jiang *et al.* showed that AST possessed antidepressant-like effects in an adult mouse model of depression (18). Acute oral administration of 20, 40, or 80 mg/kg body weight of astaxanthin 1 hr before behavioral and neurochemical tests, significantly ameliorated depressive-like behaviors with reduction in immobility time in FST and TST, but with no effect on locomotor activity (18). These results suggest that AST has neuroprotective effects against stress-induced by FST and TST. Pretreatment with para-chlorophenylalanine eliminated the anti-immobility effect of AST in FST and TST, suggesting that the mechanism of antidepressant-like effects of AST might involve the serotonergic system. Furthermore, acute treatment of AST significantly increased the noradrenaline level in the hippocampus but did not prevent reserpine-induced hypothermia and ptosis. Astaxanthin also reduced the indoleamine 2,3-dioxygenase (IDO) activity in the hippocampus, frontal cortex, and hypothalamus. This was followed by a reduction in KYN/tryptophan ratio and an increase in the serotonin/tryptophan ratio, suggesting that the antidepressant effects of AST could be mediated by the central serotonergic system (18). 

Qiao *et al.* reported that the combined treatment of lithium chloride (LiCl) and AST had synergistic antidepressant effects in a mouse model of depression induced by chronic omethoate, an organophosphorus pesticide (OP). 32 mice were subcutaneously injected with omethoate at 5 mg/kg body weight/day for 4 weeks to establish a chronic omethoate poisoned model (54). Following 4 weeks of omethoate exposure, the mice were grouped into omethoate, AST, LiCl, and combined AST-LiCl groups. The AST group was then subjected to an oral administration of AST at 50 mg/kg body weight/day whereas the LiCl group was subjected to intraperitoneal injection of LiCl at 2 mmol/kg body weight/every other day for 4 weeks. The combined AST-LiCl group received an oral administration of AST and intraperitoneal injection of LiCl. Locomotor activity and depressive-like behavior of the mice were evaluated by OFT, FST, and TST after 4 weeks of treatment (54). Astaxanthin or LiCl alone reversed the depressive-like behaviors with a reduction of immobility time in FST and TST but had no effect on locomotor activity. Nevertheless, combined administration of AST-LiCl exhibited a greater alleviation of depressive-like behavior, suggesting synergistic therapeutic effect of AST-LiCl on chronic OP-induced depressive-like behavior (54).

In addition, pretreatment with AST revealed a significant reduction in denatured cell index and nuclear pyknosis with enhanced cytoplasmic concentration in the hippocampus (54). Intriguingly, the combined treatment of AST-LiCl markedly prevented neuronal loss and decreased the denatured cell index compared with that of AST, indicating the synergistic effects of combined application. The neuroprotective effects of AST and AST-LiCl were regulated through the activation of the Akt/GSK3β/CREB signaling pathway (54). 

*Sargassum swartzii *C. Agardh, *Stoechospermum marginatum *(C. Agardh) Kützing, and *Nizamuddinia zanardinii* (Schiffner) P.C.Silva

*Sargassum swartzii, Stoechospermum marginatum, *and* Nizamuddinia zanardinii *are found in the Indian and Australian oceans as well as the Oman Sea, Yemen, and Iran. S*. swartzii* and *S. marginatum* have been reported to possess sulfated polysaccharides and fucoxanthins (74). In a study by Siddiqui *et al*., the macroalgae from the coastal region of Ormara in Pakistan were investigated for their antidepressant-like effects in a rat model of depression (28). Chronic administration of 30–60 mg/kg body weight/day of methanol extracts for 28 days significantly reduced depressive-like behaviors and immobility time in FST without locomotor impairment. A monoamine depletion paradigm was used to investigate the involvement of monoamine transmission in antidepressant effects. The antidepressant-like effect was mediated by monoaminergic modulation involving the noradrenergic-serotonergic-dopaminergic system (28).

*Padina australis *Hauck

*Padina australis *is a brown macroalga that is found in the tropical and subtropical waters of Southeast Asia (Malaysia, Japan, and Indonesia), West and East Africa, Australia, and New Zealand (75). Subermaniam *et al.* revealed that *P. australis* has the potential to attenuate high-dose (600 µM) corticosterone-mediated oxidative damage in PC12 cells mimicking the effects of depression (12). Ethanol extract of *P. australis *sampled from Cape Rachado, Malaysia was found to contain abundant phenolic and flavonoid compounds with potent anti-oxidant activities, which have been postulated to be involved in the mitigation of oxidative damage. High-dose corticosterone-induced chronic stress causing lowered glutathione (GSH) level, reduced mitochondrial membrane potential (MMP), decreased aconitase activity, increased intracellular ROS generation and accumulation, apoptosis, and cytoplasmic lactate dehydrogenase (LDH) release. However, pretreatment with the extract remarkably reversed the oxidative damage by increasing GSH level, MMP, and aconitase activity, and profoundly suppressed the intracellular ROS generation, apoptosis, and LDH release (12). Therefore, *P. australis* can be developed as a mitochondria-targeted anti-oxidant to mitigate depressive-like effects. 

*Solieria filiformis *(Kützing) P.W. Gabrielson

*Solieria filiformis *is a red macroalga found in the Mediterranean Sea, Brazil, Virginia, Britain, Australia, New Zealand, Iran, and the Persian Gulf (76). It contains sulfated polysaccharide carrageenan (55). Abreu *et al.* investigated the antidepressant- and anxiolytic-like effects of lectin isolated from *S. filiformis *(SfL) in adult Swiss mice. The mice were intravenously administered *S. filiformis* lectin (SfL) 30 min before the behavioral testing. Acute treatment of SfL at 1, 3, or 9 mg/kg body weight significantly reduced the immobility time in FST and TST, indicating an antidepressant-like effect. However, the treatment did not elicit an anxiolytic-like effect observed in the elevated plus-maze and hole-board tests or alter the locomotor activity. The mechanism underlying the antidepressant effects of SfL was analyzed by pretreating the animals with prazosin and yohimbine (noradrenergic system); SCH23390 and sulpiride (dopaminergic system); and *p*-chlorophenylalanine methyl ester and PCPA (serotonergic system). The antidepressant-like effect of SfL was blocked by pretreatment with dopamine D_1 _and D_2_ receptor antagonists, indicating involvement of the dopaminergic system (55). 

*Ulva *species

*Ulva *species are green macroalgae found in marine, fresh and brackish waters. *Ulva *species are widely distributed throughout the world with 18 species identified in Japan (77). Its cell wall consists of ulvan, a complex sulfated polysaccharide representing 8% to 29% of the dry weight (78). Violle *et al.* investigated the anxiolytic- and antidepressant-like effects of hydrophilic extract of *Ulva *sp., containing 45% of ulvan (FR 1261909) in a rat model of depression (20). Acute and subchronic oral toxicity studies showed that 10, 20, or 40 mg/kg body weight/day of hydrophilic extract of *Ulva *sp. for 14 days significantly reduced the immobility time in FST in a dose-dependent manner. A maximum effect was observed at 40 mg/kg body weight/day, which was similar to that of imipramine at 10 mg/kg body weight/day. However, the extract did not alter the behavior of the animals in open arm exploration on elevated plus-maze, suggesting no anxiolytic and anxiogenic-like effect of the extract. 

**Figure 5:**
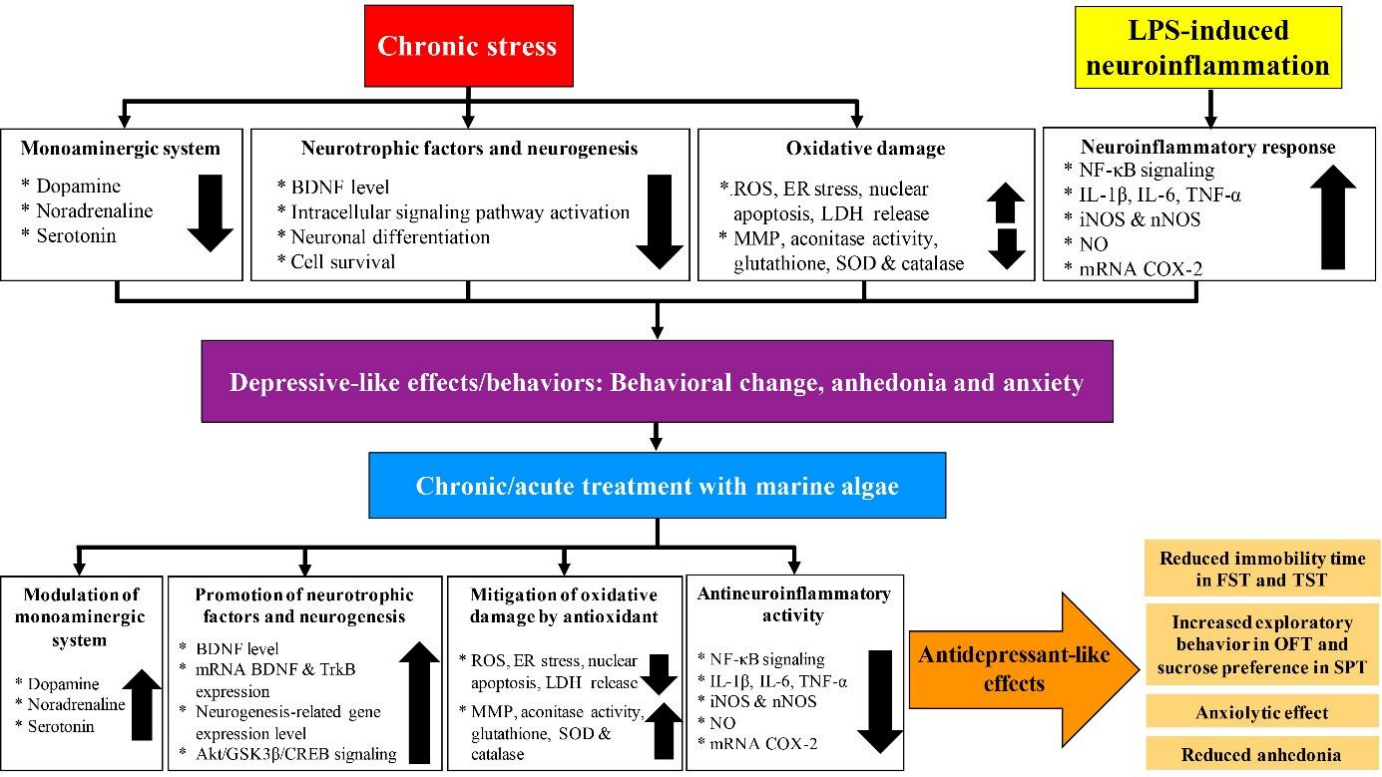
Antidepressant-like effects of marine algae against chronic stress and LPS-induced neuroinflammation in preclinical studies

Taken together, the marine algae appear to promote antidepressant-like effects by modulating the monoaminergic system and promoting neurotrophic factors, neurogenesis, anti-oxidant mechanism, and antineuroinflammatory responses (Figure 5).

***Clinical studies***

*Chlorella vulgaris* Beijerinck, M.W.

Panahi *et al.* investigated the therapeutic efficacy of a chemically defined anti-oxidant-rich *C. vulgaris* extract (CVE) (ALGOMED^®^) as an adjunct to standard treatment in patients with major depression (56). The study selected 92 patients with major depression according to the guidelines of the Diagnostic and Statistical Manual of Mental Disorders-IV (DSM-IV). The subjects were assigned to standard antidepressant therapy. 42 patients were assigned to the adjunct therapy with CVE whereas 50 patients were on standard antidepressant therapy. Participants in the CVE intervention group received six tablets of 300 mg (ALGOMED^®^) each daily for 6 weeks, totaling 1800 mg of CVE. Following 6 weeks of oral administration of CVE, the intervention group demonstrated improved somatic and cognitive symptoms of depression and anxiety, but not affective symptoms, as measured by Beck Depression Inventory-II (BDI-II) and Hospital Anxiety and Depression Scale (HADS) (56).

*Haematococcus pluvialis *Flotow

Talbott *et al.* examined the effects of astaxanthin derived from H.* pluvialis* in 28 adults diagnosed with depression and fatigue (57). The study also recruited healthy, active, and non-depressed adults (1:1 ratio of males to females). Subjects in the *H. pluvialis* intervention group received 12 mg astaxanthin (AstaZine^®^) capsule daily for 8 weeks, whereas the control group received matching placebo. Supplementation with astaxanthin significantly reduced depression and fatigue, as assessed by the Profile of Mood States (POMS) questionnaire. There are six subscales of POMS that assess the mood: tension-anxiety, depression-dejection, anger-hostility, vigor-activity, fatigue-inertia, and confusion-bewilderment (57). 

*Ulva lactuca* Linnaeus

*Ulva lactuca*, also known as *U. fenestrate* or ‘sea lettuce’, is an edible green macroalga. It is found in Europe, North America, Central America, Caribbean Islands, South America, Africa, Indian Ocean Islands, Southwest Asia, China, Pacific Islands, Australia, and New Zealand (79). Allaert *et al.* investigated the effects of *U. *lactuca water-soluble extract in adults aged 18 to 65-year-old with anhedonia (42 in the placebo group and 44 in the *Ulva* group). They reported that *U. lactuca* extract improved the neuroaffective function as assessed by the Snaith-Hamilton Pleasure Scale (SHAPS), Quick Inventory of Depressive Symptomatology-Self-Report (QIDS-SR), and Hamilton Depression Rating Scale (HAM-D) (59). Oral administration of a single dose of *U. lactuca *water-soluble extract at 6.45 mg/kg body weight/day for 3 months improved the components of depression as measured by the Patient Global Improvement Impression (PGII) and Clinical Global Improvement Impression (CGII) scales. The evaluated components of depression are anhedonia, sleep disorder, psychomotor consequences, and nutrition behavior (59). 

***Undaria pinnatifida (wakame), Sargassum fusiforme (hijiki), and Pyropia yezoensis, Pyropia tenera (nori)***

*Undaria pinnatifida*, a brown macroalga also known as wakame, is found in Japan, Korea, China, New Zealand, United States, Belgium, Australia, Spain, Italy, Mexico, and France (76). *Sargassum fusiforme*, a brown macroalga also known as hijiki, is found in Japan, Korea, China, and Hong Kong (80). *Pyropia, *a red macroalga commonly used to produce nori, is found in Japan, Korea, and China (81). Miyake *et al.* studied the clinical effects of nori and wakame or nori and hijiki on symptoms of depression during pregnancy through a self-administered questionnaire in 1745 pregnant women between the 5^th^ and 39^th^ week of pregnancy (58). Baseline data from the Kyushu Okinawa Maternal and Child Health Study (KOMCHS) was employed, and symptoms of depression were assessed by the Center for Epidemiologic Studies Depression Scale (CES-D). Daily consumption of selected nutrients was estimated using an *ad hoc* computer algorithm following a diet history questionnaire (DHQ) according to the Standard Tables of Food Composition. The prevalence of symptoms of depression was reduced with higher consumption of macroalgae, suggesting the potential therapeutic efficacy of macroalgae in the treatment of depression (58).

*Other macroalgae*

A prospective cohort study conducted between 2008 and 2011, revealed a link between higher macroalgae consumption and a lower incidence of symptoms of depression among 500 working Japanese adults aged 20 to 74 years (60). The consumption was divided into three categories as decreased group (decreased by < 2 g/1000 kcal/day), unchanged group (changed from -2 to 2 g/1000 kcal/day), and increased group (increased by > 2 g/1000 kcal/day). Symptoms of depression were assessed by the Self-Rating Depression Scale (SDS) and defined as SDS score of ≥ 50. The odds ratio for symptoms of depression were lower in participants of the “increased group” than the “decreased group”. The association was independent of sex, age, body mass index, socioeconomic status, lifestyle, and intake of other food items. Nevertheless, 46 or 9.2% of participants exhibited symptoms of depression during the study (60).

**Lmitations and future directions**

We reviewed 17 studies that investigated the use of 14 marine algae in the treatment of depression. Besides the normal widespread consumption of marine algae, animal studies and anecdotal clinical experiences also reported potential antidepressant effects of marine algae. However, such observations do not replace well-designed randomized controlled trials, the gold standard for investigating the safe clinical therapeutic effects of natural products. Randomized clinical trials including single- or double-blind placebo-controlled studies are considered to be crucial for providing robust scientific evidence to support the efficacy of natural products tested in preclinical trials (82). In the current investigations, the majority of the clinical studies were observational in design. Therefore, randomized controlled trials (RCT) are still needed to verify the efficacy of marine algae in eliciting antidepressant-like and anxiolytic-like effects before translating their application to daily clinical use for treating patients with depression. 

The majority of these studies focused on examining the antidepressant effects of extracts from marine algae. Although the studies on marine algae provide evidence of their potential antidepressant-like effects, the precise underlying mechanisms have yet to be examined. Marine algae have been reported to have substantial bioactive metabolites and compounds with great pharmaceutical and biomedical potential (14, 51). It is necessary to investigate the efficacy of the isolated bioactive compound(s) and their underlying neurobiochemical mechanisms of action before developing them into a novel antidepressant agent. This is to ensure the absence of adverse effects compared with conservative or synthetic antidepressants which often cause side effects. 

To elucidate the mechanism underlying the antidepressant effects of hydrolyzed *Spirulina *by malted barley, Kim *et al*. examined the biochemical parameters in mice after FST, as the decreased duration of immobility could be due to changes in certain metabolites (34). However, the metabolic changes attributed to this behavioral result were not clearly displayed in this study. The observed antidepressant effects may also have been mediated by the synergistic effect of compounds in the malted barley, hence, a more precise metabolic study is needed to determine the association of the observed antidepressive-like behavior with hydrolyzed *Spirulina* by malted barley. In addition, a study examining the antidepressant effects of hydrolyzed *Spirulina *alone is recommended to further validate the efficacy of *Spirulina*. Further, it would be interesting to investigate the accurate metabolic activity of blood urea nitrogen and lactate dehydrogenase to better understand the antidepressant response as indicated in the study. 

Although *S. platensis *was postulated to alleviate the symptoms of depression by stimulating the monoaminergic system, an examination of its role as an agonist or inhibitor of monoamine neurotransmitter receptors is still lacking. It would also be interesting to conduct a clinical study to investigate the role of the bioactive compounds attributed to its antidepressant effects (52). Although Moradi-Kor *et al.* demonstrated that *S. platensis* is a promising antidepressant agent, their conclusions are less convincing, as the study was female gender-specific (21), similar to the study design of Soetantyo and Sarto (53). Hormonal changes that occur in the estrus cycle of females have been associated with alterations in neuronal and behavioral manifestations (83). Future studies need to address the lack of sex hormone measurements during the estrus cycle to elucidate their influence on the efficacy of *S. platensis* and *C. vulgaris* (21, 53). Is necessary to examine the antidepressant effects of the algae in male rats to eliminate the possibility that female hormonal changes affect neuronal or behavioral indices. Furthermore, the effects of *C. vulgaris* on physiological changes in the study by Soetantyo and Sarto are less convincing because of the short treatment period, which might not provide enough time for structural changes to develop (53). In addition, *C. vulgaris* is rich in carbohydrates in the form of sugars including galactose, glucose, and mannose (68). Cultivated and commercial microalgae treatments are found to increase blood glucose levels, reducing their suitability as antidepressants for the population. 

In the study by Violle *et al.*, the antidepressant properties of the hydrophilic extract of the *Ulva *sp. were associated with immunomodulatory effects of ulvan (sulfated polysaccharides) on the gastrointestinal tract (20). However, investigating the precise underlying molecular mechanism of action of the hydrophilic extract of ulvan on modulating brain function would be more appropriate. In the study by Sasaki *et al.*, *B. braunii *was shown to exhibit antidepressant effects in an animal model of depression induced by FST (19). This test is a well-known animal behavioral test and is generally accepted in the study of depression (84). However, despite promising results, the experimented animals were not pre-exposed to stress prior to the treatment with *B. braunii* ethanol extract and were exposed to FST for a duration of 5 min for only 5 days in 14 days of the treatment. Therefore, this raises the question of the efficacy of *B. braunii, *which may vary between stress-exposed and non-stress-exposed animal models. Establishing a chronic stress animal model of depression is important and pretreating the animals prior to chronic stress would yield more convincing results (84).

In the study by Jiang *et al.*, astaxanthin was shown to demonstrate antidepressant-like effects via modulation of the inflammatory pathway, but the therapeutic effect of the compound remains unanswered (17). The clinical treatment of depression requires the long-term administration of antidepressants to obtain a therapeutic effect, hence 7 days of treatment may not be sufficient to produce the desired outcome. Jiang *et al.* demonstrated the antidepressant effects of astaxanthin in a behavioral despair test (18). However, the use of an acute or a single dose treatment may not be able to elicit long-term therapeutic effects, thus increasing the risk of recurrence (9). Further experiments with longer duration of astaxanthin treatment would provide a better understanding of its pharmacotherapeutic potential as a therapy for depression. The study by Qiao *et al.* involved the long-term administration of astaxanthin (54), which showed similar antidepressant effects to the study by Jiang *et al.* (18) in which only acute dosing of astaxanthin was given. Therefore, further comparison research on the antidepressant effects of acute and long-term astaxanthin treatment is needed to clarify the need for long-term treatment in depressive disorders. In addition, Qiao *et al.* showed that pretreatment with astaxanthin alone displayed remarkable antidepressant effects, thus the need for LiCl to produce a synergistic therapeutic impact on OP-induced depressive-like impairment is debatable (54).

Although Siddiqui *et al.* showed that the antidepressant effects generated by *S. swartzii, S. marginatum,* and *N. zanardinii* involved the monoaminergic system, the synergistic interaction between neurotransmitters makes it difficult to determine which single neurotransmitter was responsible for the observed response (28). Having therapeutic effects found for all three species would be great treatment options for depression. Nevertheless, further investigations of the intracellular signaling mechanism between the neurotransmitters on the antidepressant effects of these extracts are needed to better understand the precise mechanism of the monoaminergic modulation. The induction of chronic stress is a well-known method in preclinical studies of depression and the establishment of an animal model of depression in the study would validate the antidepressant effects of the tested species. In addition, a chemical analysis is needed to elicit the exact compound(s) responsible for the observed effects.

The results from the study by Abreu *et al*. on the antidepressant effects of SfL are less convincing as the animal model of depression was established using naive animals that were not pre-exposed to stress, and thus the effects in depressed subjects are not known (55). The efficacy of the SfL as an antidepressant agent may vary between naive and depressed subjects as they may have different behaviors and physiological responses. As mentioned previously, treatment of depression usually requires a long-term administration of antidepressant drugs and acute treatments may not provide long-term therapeutic effects, thus increasing the risk of recurrence (9). In addition, a study investigating the use of a serotonergic antagonist on the observed effect of SfL is needed to validate the antidepressant effects of this marine alga. Although the anti-oxidants derived from *P. australis *were shown to attenuate depressive-like effects in the study by Subermaniam *et al.*, the precise mechanism of the observed effects was not reported (12). Among many other hypotheses, dysregulation of the hypothalamic-pituitary-adrenal axis is the most prominent neurobiological change in the etiology of depression (85). Further studies examining the efficacy of *P. australis*-derived anti-oxidants on HPA axis dysregulation-induced oxidative damage are highly warranted to better understand the mechanism by which *P. australis* exerts its antidepressant effects (12).

A cross-sectional study by Miyake *et al.* found that higher macroalgae consumption during pregnancy was independently associated with lower prevalence of symptoms of depression (58). Nevertheless, the dietary history questionnaire provided only an approximation of the consumption of the three types of macroalgae. Furthermore, the observed results may have been biased owing to non-differential exposure misclassification during pregnancy, as the study subjects were at various points of their pregnancy and, the exact incidence and prevalence of symptoms of depression during pregnancy were not known. The scale used to assess the symptoms of depression included physical symptoms that are also typical manifestations experienced during pregnancy, thus the overlapping symptoms may have led to an overestimation of the prevalence of depression. The participation rate was not calculated as there were problems in obtaining an accurate number of pregnant women who were given study documents. Furthermore, the sociodemographic characteristics of the subjects might not represent the general population. Considering the tested seaweed species are all edible, a randomized controlled trial would be an ideal method to attribute causality in relation to macroalgae consumption and prevalence of symptoms of depression during pregnancy (58). 

Panahi *et al. *reported that the adjunct therapy with *C. vulgaris* was efficacious and safe in the majority of depressive patients. Although it was shown to improve physical and cognitive symptoms of depression and anxiety symptoms, they conducted only a short-term follow-up assessment (56). Considering major depression is a chronic disorder, a long-term treatment with long-term follow-up is essential to provide convincing evidence of the *C. vulgaris*’s efficacy as an adjunct therapy. Moreover, the heterogeneity of standard antidepressants taken in combination with *C. vulgaris* may have biased the observed effects of the adjunct treatment. Hence, further investigations assessing the differential effects of *C. vulgaris *in combination with specific classes of antidepressant drugs are strongly recommended (56). In addition, open-label trials are inferior to double-blind and placebo-controlled clinical trials in determining the efficacy of an intervention and to minimize assessment bias (86). The potential use of *C. vulgaris* as an adjunct therapy in patients with major depression may be further enhanced in future double-blind studies with larger populations and longer follow-up. 

The efficacy of* U. lactuca* extracts in improving anhedonia, a component of depression, in healthy volunteers has been demonstrated in a randomized placebo-controlled double-blind clinical trial by Allaert *et al.* (59). It would be interesting to examine compounds isolated from *U. lactuca* extract to identify the specific active ingredient attributed to the anti-anhedonia effects. Although astaxanthin supplementation was shown to reduce depression and fatigue in healthy subjects in the study by Talbott *et al.,* the inclusion criteria of the subjects may lead to contradictory results (57). The reduction in the symptoms of depression in healthy subjects after 8 weeks of astaxanthin supplementation is questionable because it is unlikely that healthy subjects would develop depression within 8 weeks without stimulation. Moreover, the results were based on self-reported questionnaires, which may be biased by the participants’ responses. Thus, further studies or trials are needed to properly examine the efficacy of astaxanthin as an adjunct therapy in promoting antidepressant effects in depressive patients. 

A 3-year prospective cohort study showed that dietary intake of macroalgae led to a lower incidence of symptoms of depression in adults (60). However, a change to higher intake of macroalgae could have been impacted by the 2011 Great East Japan Earthquake, which happened 5 months before the follow-up study, or the Fukushima nuclear accident disasters, and this could have potentially confounded the results. In general, the possibilities are that the Japanese population could have taken less seafood than before due to the risk of agricultural and fishery products being contaminated owing to the Fukushima nuclear accident. On the other hand, some of the participants could have consumed more macroalgae after the disasters with an intention to increase their iodine intake for the therapeutic effect on thyroid diseases (87). In addition, information relating to macroalgae was not reported, and it is not possible to attribute the antidepressant effects to a specific species. Different macroalgae have different nutritional values depending on their species, and hence have different health benefits (88). Moreover, the amount of macroalgae consumption was self-reported by the study subjects, thus the reduction in the symptoms of depression in relation to the amount of algae consumed may have been underestimated or overestimated. The cooking method was also not indicated in the questionnaire and therefore, the nutritional value of algae consumed may differ between subjects due to sensitivity of different classes of nutrients toward different cooking methods. As this was an observational study, the causality could not be determined. A randomized controlled trial using a single edible species of macroalgae is needed to examine the relationship between macroalgae intake and the symptoms of depression (60).

In this review, we summarized the extensive experimental and clinical studies of numerous marine algae as promising adjunctive agents for treating depression. Current synthetic antidepressant medications usually have side effects or are slow to exert their antidepressant effects (89) which can lead to high rates of antidepressant treatment discontinuation or nonadherence (90). The use of natural products as an alternative therapy for depression, particularly those with fewer side effects, is gaining wider acceptance. Numerous natural products have been traditionally used as alternative medicines for treating mood disorders (91). Recent studies have indicated the interaction of oxidative stress and inflammation in the pathophysiology of depression (92, 93). Phytochemicals from natural products represent a huge source of active compounds for the discovery of new and more effective antidepressant drugs. Identifying the active compounds in marine algae and improving the extraction methods would be necessary to improve the efficacy of the respective species to facilitate the development of treatments for depressive disorders. Although the consumption of marine algae or their extracts and compounds have shown promising antidepressant and anxiolytic effects, there is still a lack of convincing evidence with regards to the ability of marine algae to efficiently ameliorate depression and anxiety in preclinical and clinical studies, warranting upcoming robust studies in this area. Furthermore, the mechanism of action of the antidepressant effects exerted by marine algae requires further investigation in order to translate these novel antidepressants into clinical practice.

**Table 1:** Antidepressant effects of marine algae examined in preclinical and clinical studies of depression

**Microalgae/** **Macroalgae**	**Algae/ Extract/ Compound **	**Dosage and treatment**	**Research model**	**Model establishment**	**Anatomical region **	**Behavioral outcome**	**Morphological/ biochemical findings**	**Authors**
**Preclinical studies**								
*Arthrospira platensis *Gomont *(Spirulina platensis)*	Hydrolyzed *Spirulina *extract by malted barley	10 ml/kg body weight/day for 2 weeks; PO	Male ICR mice	Exposure to FST	N.A.	FST: reduced immobility time	Reduced plasma levels of blood urea nitrogen and lactate dehydrogenase	(34)
	Spray-dried powder	100, 200, and 400 mg/kg body weight/day for 1 week; PO	Wistar albino rat and albino mice	Exposure to FST, TST, 5-HTP-induced head twitches, clonidine-induced aggression, L-DOPA-induced hyperactivity, and aggressive behavior	Brain	FST: reduced immobility time TST: reduced immobility time 5-HTIP-induced head twitches: increased Clonidine-induced aggression: increased L-DOPA-induced hyperactivity and aggressive behavior: increased	Increased SOD and catalase levels in the brain Toxicity at the maximum dose (2000 mg/kg) of *spirulina *	(52)
	Pure powder (SPM-12462016)	200 mg/kg body weight/day for 15 days; PO	Wistar female rats	Restraint stress	Basolateral amygdala	N.A.	Decreased SOD and GPx activity Increased FRAP level Decreased MDA level Increased BDNF level in amygdala Increased BDNF and TrkB mRNA expression Increased apical dendritic length and number of dendritic spines of basolateral amygdala neurons	(21)
*Botryococcus braunii *Kützing	Ethanol extract	100 mg/kg body weight/day for 2 weeks; PO	Male ICR mice	Exposure to FST	Cerebrum	FST: reduced immobility time	Increased ATP production Up-regulated mRNA expression of tyrosine 3-monooxygenase, pyruvate carboxylase, and BDNF Induced dopamine synthesis- and neurogenesis-related gene expression	(19)
		1/1000 (0.1%) dilution of ethanol extract for 48 h; *in vitro*	PC12 cells	Exposure to corticosterone	N.A.	N.A.	Increased cell viability	
*Chlorella vulgaris *Beijerinck, M.W.	*C. vulgaris* powdered extract and commercialized *C. vulgaris* tablet	360 mg/kg body weight/day for 2 weeks; PO	Wistar rat	Chronic unpredictable mild stress	Adrenal gland	FST: reduced immobility time OFT: increased motor activity SPT: increased sucrose preference	N.A.	(53)
*Haematococcus pluvialis *Flotow	Astaxanthin Astaxanthin Astaxanthin Combined astaxanthin-lithium chloride	20, 40, and 80 mg/kg body weight/day for 7 days; PO	Male ICR mice	Exposure to IP injection of lipopolysaccharide	Hippocampus, prefrontal cortex	FST: reduced immobility time TST: reduced immobility time	Reduced pro-inflammatory cytokines (IL-1β, IL-6, TNF-a), inflammatory mediators (iNOS, nNOS, and COX-2 mRNA expression) Suppressed NF-κB p65 phosphorylation	(17)
		Single dose of 20, 40, and 80 mg/kg body weight; PO	Male ICR mice	Exposure to FST and IP injection of *p*-chlorophenylalanine and reserpine	Frontal cortex, hippocampus, hypothalamus, striatum	FST: reduced immobility time TST: reduced immobility time	Increased serotonin level in all tested brain regions Increased noradrenaline in the hippocampus Decreased indoleamine 2, 3-dioxygenase mRNA expression Reduced kynurenine/ tryptophan ratio Increased serotonin/tryptophan ratio in the hippocampus, frontal cortex, and hypothalamus	(18)
		50 mg/kg body weight/day for 4 weeks; PO Astaxanthin 50 mg/kg body weight/day for 4 weeKS; PO and lithium chloride 2 mmol/kg body weight/every other day for 4 weeks; IP	Adult male Kunming mice	Chronic exposure to organophosphorus pesticides	Hippocampus (dentate gyrus)	FST: reduced immobility time TST: reduced immobility time	Reduced neuronal damage in the hippocampus Increased p- GSK3β, p-CREB, p-P13K and p-Akt expressions in the hippocampus Activated Akt/GSK3β/CREB signaling pathway	(54)
*Nizamuddinia Zanardinii *(Schiffner) P.C.Silva	Methanol extract	30, 45, and 60 mg/kg body weight/day for 4 weeks; PO	Male Wistar albino rat	Exposure to FST	Brain tissues	FST: reduced immobility time	Increased brain monoamine levels (serotonin, dopamine, and noradrenaline)	(28)
*Padina australis *Hauck	Ethanol extract	Single dose of 0.25–0.5 mg/ml for 24 hr; *in vitro*	PC12 cells	Exposure to high dose corticosterone	N.A.	N.A.	Reduced lactate dehydrogenase release, intracellular reactive oxygen species, endoplasmic reticulum stress, and apoptosis Increased cell viability, endogenous glutathione, mitochondrial membrane potential, and aconitase activity	(12)
*Stoechospermum marginatum *(C.Agardh) Kützing	Methanol extract	30, 45, and 60 mg/kg body weight/day for 4 weeks; PO	Male Wistar albino rat	Exposure to FST	Brain tissues	FST: reduced immobility time	Increased brain monoamine levels (serotonin, dopamine, and noradrenaline)	(28)
*Sargassum swartzii* C. Agardh	Methanol extract	30, 45, and 60 mg/kg body weight/day for 4 weeks; PO	Male Wistar albino rat	Exposure to FST	Brain tissues	FST: reduced immobility time	Increased brain monoamine levels (serotonin, dopamine, and noradrenaline)	(28)
*Solieria filiformis (*Kützing) P.W. Gabrielson	*Solieria filiformis *lectin	Single dose of 1, 3, and 9 mg/kg body weight; IV	Male adult Swiss mice	Exposure to FST	N.A.	FST: reduced immobility time TST: reduced immobility time Interaction with the dopaminergic system possibly via D_1_- and D_2_- receptors		(55)
*Ulva *sp.	Hydrophilic extract (lyophilized powder) (FR 1261909)	10, 20, and 40 mg/kg body weight/day for 2 weeks; PO	Male and female Wistar rats	Exposure to FST	N.A.	FST: reduced immobility time	N.A.	(20)
**Clinical studies**								
*Chlorella vulgaris *Beijerinck, M.W.	*C. vulgaris* extract	300 mg/tablet (ALGOMED^®^) 3 times/day for 6 weeks; PO	Adult patients with major depression	N.A.	N.A.	Physical and cognitive symptoms of depression: improved Anxiety symptoms: improved	N.A.	(56)
*Haematococcus pluvialis *Flotow	Astaxanthin	12 mg/capsule (AstaZine^®^)/ day for 8 weeks; PO	Healthy adults	N.A.	N.A.	Negative mood state (depression and fatigue): reduced Global mood state: improved	N. A.	(57)
*Sargassum fusiforme* and *Pyropia yezoensis *or *Pyropia tenera *	Whole macroalgae	Consumption ranging from at least twice/day to less than once/month; PO Portion size ranging from very small (50% or less) to very large (50% or more) of a standard portion size	Pregnant women (5 to 39 weeks pregnancy)	N.A.	N.A.	Prevalence of symptoms of depression: reduced with higher consumption of macroalgae	N.A.	(58)
*Ulva Lactuca *Linnaeus	Water-soluble extract	6.45 mg/kg body weight /day in the form of capsules for 12 weeks; PO	Adults with anhedonia	N.A.	N.A.	Anhedonia: reduced Sleep disorder: improved Psychomotor consequences: improved Nutrition behavior: improved	N.A.	(59)
*Undaria pinnatifida* and *Pyropia yezoensis *or *Pyropia tenera*	Whole macroalgae	Consumption ranging from at least twice/day to less than once/month; PO Portion size ranging from very small (50% or less) to very large (50% or more) of a standard portion size	Pregnant women (5 to 39 weeks pregnancy)	N.A.	N.A.	Prevalence of symptoms of depression: reduced with higher consumption of macroalgae	N.A.	(58)
Unnamed	Whole macroalgae	Consumption ranging from almost never/day to twice or more times/day for 3 years; PO	Adult employees	N.A.	N.A.	Incidence of symptoms of depression: reduced with higher consumption of macroalgae	N.A.	(60)

PO = per oral, IV = intravenous injection, IP = intraperitoneal injection, SOD = superoxide dismutase, GPx = glutathione peroxidase, FRAP = ferric reducing antioxidant power, MDA = malondialdehyde, BDNF = brain-derived neurotrophic factor, TrkB = tyrosine receptor kinase B, ATP = adenosine triphosphate, FST = forced swim test, TST = tail suspension test, SPT = sucrose preference test, Akt = protein kinase B, GSK3β = glycogen synthase kinase 3 beta, CREB = cAMP-response element binding protein, 5-HTP = serotonin, IL-1β = interleukin-1 beta, IL-6 = interleukin-6, TNF-α = tumor necrosis factor-alpha, iNOS = inducible nitric oxide synthase, nNOS = neuronal nitric oxide synthase, COX-2 = cyclooxygenase-2, NF-κB = nuclear factor kappa B, N.A.: Not applicable

## Conclusion

Although the use of marine algae as a treatment option for depression is still considered to be in the early stages, their promising antidepressant properties observed in the preclinical and clinical trials are generating much interest. Natural products that have fewer side effects are generally favored compared with synthetic antidepressant drugs that may have severe adverse effects. The reviewed studies together with the first *in vitro* study of the antidepressant effects of marine algae-derived anti-oxidants have provided us a promising outlook of the potential use of marine algae as novel antidepressants in clinical practice. Perhaps, the application of marine algae as an adjunct therapy in combination with standard antidepressants may encourage the development of marine algae-based antidepressants in the near future. Nevertheless, it is crucial to ensure these potential marine algae-based therapeutics conform to the required standards of safety, quality, and efficacy according to drug regulatory frameworks. 
